# Blood mRNA biomarkers distinguish variable systemic and sputum inflammation at treatment initiation of inhaled antibiotics in cystic fibrosis: A prospective non-randomized trial

**DOI:** 10.1371/journal.pone.0267592

**Published:** 2022-05-05

**Authors:** Silvia M. Caceres, Linda A. Sanders, Noel M. Rysavy, Katie R. Poch, Caroline R. Jones, Kyle Pickard, Tasha E. Fingerlin, Roland A. Marcus, Kenneth C. Malcolm, Jennifer L. Taylor-Cousar, David P. Nichols, Jerry A. Nick, Matthew Strand, Milene T. Saavedra

**Affiliations:** 1 Department of Medicine, National Jewish Health, Denver, Colorado, United States of America; 2 Department of Medicine, University of Colorado Denver, Aurora, Colorado, United States of America; 3 Center for Genes, Environment, and Health, National Jewish Health, Denver, Colorado, United States of America; 4 Department of Pediatrics, Children’s Hospital Colorado, Aurora, Colorado, United States of America; 5 Department of Pediatrics, University of Washington, Seattle, Washington, United States of America; 6 Division of Biostatistics, National Jewish Health, Denver, Colorado, United States of America; Prince Sattam Bin Abdulaziz University, College of Applied Medical Sciences, SAUDI ARABIA

## Abstract

Inhaled antibiotics control chronic airway infection and maintain respiratory health in cystic fibrosis (CF). Given variation in patient responses to inhaled antibiotics, the ability to identify distinct responder phenotypes would facilitate the delivery of personalized care. Previously, a 10-gene panel was identified, measured directly from blood leukocytes, which predicted host response to intravenous antibiotic treatment during pulmonary exacerbations. In the current study, we tested whether the same panel predicted clinical response in subjects receiving a month of inhaled antibiotic therapy with aztreonam lysine (AZLI; Cayston®). A small cohort of CF subjects infected with *Pseudomonas aeruginosa* were enrolled at baseline health, prior to initiating one month’s treatment with AZLI using the Altera® nebulizer system. Eighteen CF subjects underwent blood leukocyte gene panel measurements, sputum quantitative microbiology, spirometry, and C-reactive protein (CRP) measurement prior to onset and at completion of 4 weeks of AZLI therapy. Mean absolute improvement in percent predicted Forced Expiratory Volume in one second (ppFEV_1_) was 3%. Significant reductions in sputum bacterial colony counts were detected with treatment. CRP increased following treatment. While single genes within the panel did not change significantly following treatment, the analysis of multigene panel data demonstrated that *HCA112* gene predicted ppFEV_1_ improvement. Hierarchical clustering based on gene expression yielded two distinctive molecular clusters before and after AZLI therapy. In conclusion, peripheral blood leukocyte genes quantifying inflammation are associated with responses to inhaled antibiotic therapy. Molecular quantification of systemic inflammation may indicate subgroups of CF subjects with variations in underlying inflammation and with variable clinical responses to inhaled antibiotics. Given the size limitation of the study, larger studies are needed in order to evaluate whether molecular measures may add precision to the determination of infectious and inflammatory outcomes following courses of inhaled antimicrobial therapies. Clinical Trials.gov Identifier: NCT01736839.

## Introduction

The use of inhaled antibiotics as chronic suppressive therapy has been a cornerstone of infection management in cystic fibrosis (CF) for patients infected with *Pseudomonas aeruginosa* (PA). Yet, the natural history of CF is rapidly evolving, as newer highly effective modulator therapies have become widely available for the US CF population. Based on partial restoration of CFTR (CF transmembrane conductance regulator) activity, elexacaftor-tezacaftor-ivacaftor markedly improves pulmonary function and reduces frequency of CF pulmonary exacerbations [1, 2]. Its introduction into the CF armamentarium signals newer challenges in managing chronic respiratory infections over a longer lifespan. Going forward, it will be important to identify individualized patient responses to inhaled antibiotic regimens to optimize response and minimize antimicrobial resistance. Based on the stabilizing effect of modulator therapy on lung disease progression, FEV_1_ may be a less sensitive marker of response, and thus changes in spirometry less detectable following inhaled antibiotic administration [3–5].

Chronic airway infection is common in adult CF patients. PA infection, although declining, remains a primary pathogen in adult CF patients in the US [6]. The use of inhaled antibiotics to suppress PA has become standard care for CF patients in the United States since the FDA approval of Tobramycin inhalation solution (TIS) in 1998 [7, 8]. Current FDA approved anti-pseudomonal nebulized antibiotics include Tobramycin inhalation solution (available as TOBI®, Novartis A.G. or Bethkis®, Chiesi Pharmaceuticals) and aztreonam lysine inhaled solution (AZLI; Cayston®; Gilead Sciences), a lyophilized formulation of the monobactam antibiotic aztreonam [9]. Both inhaled antibiotics are often utilized in an alternating on-off month cycle, which has become an increasingly common trend in CF clinical care [9–13]. Their treatment benefits include a mean improvement in pulmonary function, reduced *Pseudomonas spp*. burden, reductions in pulmonary exacerbations, and greater quality of life for study populations [7, 9]. Variability of responses to these therapies is substantial. Thus, in AZLI Phase 3 studies, mean adjusted relative FEV_1_ improvement was 10.3%, whereas the confidence interval ranged from 6.3 to 14.3%, with smaller FEV_1_ improvements seen in subjects with more severe pulmonary disease [9].

To better understand whether differences in host inflammation were associated with variable response, we hypothesized that a previously identified blood leukocyte gene panel could identify variable treatment response to inhalational aztreonam following a month of therapy. The panel categorizes changes in systemic inflammation during CF pulmonary exacerbation treatment, even with small reductions (0.3 log) in bacterial density [14]. It enhances the accuracy of FEV_1_ to identify reductions in airway infection and in particular was highly specific to detect reduced infection [14]. We performed a prospective study on a CF cohort at baseline health, monitoring gene panel measurements, spirometry, quantitative sputum microbiology, CRP and sputum neutrophil elastase before and after one month of AZLI.

## Methods

The single site study was approved by the National Jewish Health Institutional Review Board (HS-2656). Written consent was obtained from participants. The inclusion criteria included: CF patients of age ≥18 years, baseline health status, infection with *P*. *aeruginosa*, ability to expectorate sputum, and perform spirometry. FEV_1_ criteria for enrollment was consistent with the FDA label for treatment with AZLI (FEV_1_ >25% and <75% predicted).

### Treatment protocol

**Fig 1:**
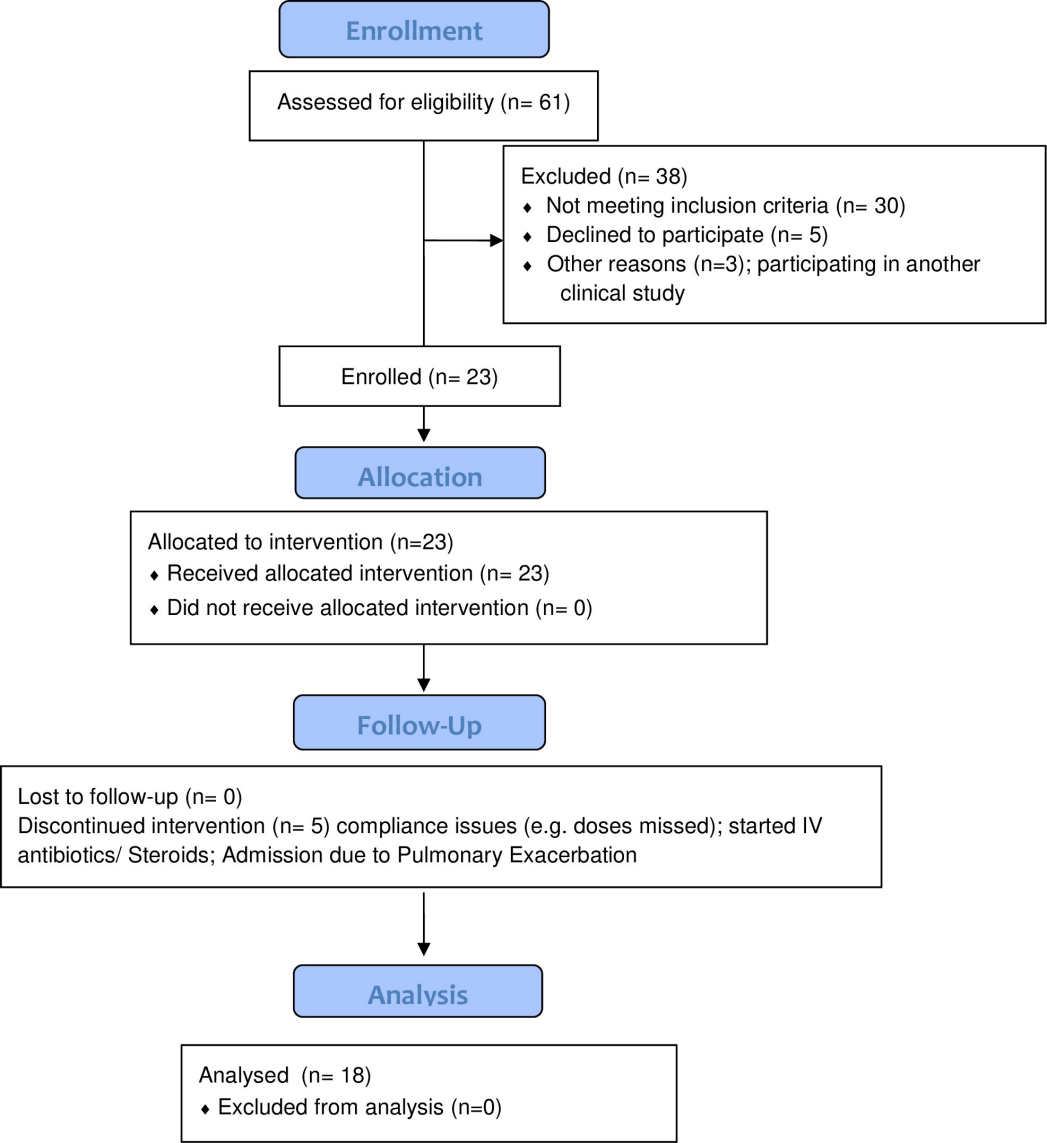
Study CONSORT flowchart.

Subjects underwent a one-month washout of all inhaled and intravenous antibiotics prior to starting AZLI treatment. Subjects then initiated a one-month treatment cycle, as per FDA label, nebulizing 75 mg three times daily. Subjects underwent phlebotomy, spirometry, sputum collection, and assessment of quality of life with CFQ-R survey before and after AZLI treatment at the Clinical Research Unit in National Jewish Health. Spirometry was performed according to American Thoracic Society guidelines [15]. Flow of participants through the study is shown in Fig 1.

### Sputum testing

Sputum samples were collected pre- and post-treatment to quantify microbiology and neutrophil elastase (NE). Quantitative microbiology culture methods conformed to the CFF Guidelines Statement for Microbiology and Molecular Typing [16]. Sputum samples were processed following the Cystic Fibrosis Therapeutic Development Network (CFTDN) standard operating procedures for cytology and inflammatory markers [17]. All supernatants were aliquoted and stored at -80°C.

Neutrophil Elastase activity was quantified by spectrophotometry assay, previously reported [18]. Briefly, a sputum aliquot alongside standards for human sputum leukocyte elastase (EPC, INC) was plated in a 96 well plate. Hydrolysis of substrate solution MeO-suc-Ala-Ala-ProAla-p-nitroanilide (Sigma-Aldrich) was measured kinetically in a microplate reader every 20 seconds up to 5 minutes at a wavelength of 405 nm. Results were normalized to the initial weight of the sputum sample.

### Measurement of leukocyte RNA from whole blood

Whole blood was collected into PAXgene^TM^ Blood RNA tubes (PreAnalytiX, Switzerland) in order to measure a previously identified 10-gene panel [14, 19]. RNA purification and first strand cDNA synthesis were fully automated. Transcript abundance was quantified by RT-PCR in pre- and post-antibiotic samples for the following 10 genes: *CD36*, *CD64* (also known as *FCRG1A*), *CD163*, Toll like receptor 2 (*TLR2*), plexin D1 (*PLXND1)*, hepatocellular carcinoma antigen 112 (*HCA112*, also known as transmembrane protein 176A, *TMEM176A*), heparanase (*HPSE)*, a disintegrin and metalloproteinase domain 9 (*ADAM9)*, versican *(CSPG2*, also known as *VCAN*) and *IL-32α*. Each targeted gene measurement was made in triplicate and expressed relative to the detection of the housekeeping gene, hypoxanthine guanine phosphoribosyl transferase (*HPRT*).

### Sample size and statistical analysis

A power analysis indicated that a sample size of 28 subjects would be required to yield 80% power to detect clinically significant mean changes in the expression of a single gene pre- and post- inhaled antibiotic therapy. Descriptive statistics were used to characterize subject demographics. Data distributions were tested using a Shapiro-Wilk test. Right-skewed variables (all except ppFEV_1_, *CD63* and *TLR2*) were natural-log transformed before analysis. Paired t-tests were used to compare gene expression variables, ppFEV_1_, percent predicted forced expiratory flow at 25 to 75% of the pulmonary volume (ppFEF_25-75_)_,_ CRP, bacterial counts and neutrophil elastase between pre and post-treatment time points for all subjects, and for cluster subgroups identified in the hierarchical clustering analysis. Two independent sample t-tests were used to evaluate differences in variables between cluster subgroups for the following: CRP, neutrophil elastase, FEF_25-75_, FEV_1_, gram negative bacteria, and the 10 gene variables. Nonparametric tests (Wilcoxon signed-rank for paired data and Wilcoxon rank-sum for 2-sample data) were used for confirmation. A linear mixed model was used to predict FEV_1_ outcomes using selected sets of predictors, that included gene variables. A random intercept term for subjects was included to account for 2 measures per subject. Predictors were selected based on initial screening using stepwise selection, and then using backward selection and Akaike’s information criterion (AIC) goodness-of-fit statistic for final selection. Ward’s linkage hierarchical clustering was performed in order to cluster subjects and variables with respect to gene expression, separately for pre- and post-treatment time points. Statistical analyses were conducted with Prism 8.1, R 3.6.1, and SAS 9.4.

## Results

The characteristics of the study population are shown in Table 1. A total of 23 subjects were enrolled, and 18 subjects completed all study procedures during a 2-year period. Enrollment was limited at the time and did not achieve the planned sample size. Therefore, our results are limited to our small size cohort, and the analyses should be considered exploratory.

**Table 1:** Characteristics of study population.

**Number of subjects**		18
**Age in years** ^**a**^		32± 10
**Female, n (%)**		10 (56)
**Genotype, n (%) F508del/F508del**		9 (50)
**Race, ethnicity, n (%)**		
Non-Hispanic white		18 (100)
**CFQ-R respiratory score** ^**a**^		
Pre-treatment		62.6 ± 14.4
Post-treatment		66.7 ± 18.1
**FEV**_**1**_ **(% predicted)** ^**a**^^**b**^		
Pre-treatment		57± 17
Post-treatment		61 ± 18
**Change in absolute ppFEV1 (range)**		-1.8 to 11
**CRP (mg/dL)** ^**a**^^**c**^		
Pre-treatment		0.48 ± 0.54
Post-treatment		0.92 ± 1.1
**Neutrophil Elastase from Sputum** ^**a**^ **(μg/mL)**		
Pre-treatment		10.12 ± 12.44
Post-treatment		14.29 ± 18.70
**CFRD, n (%)**		7 (39)
**Microbiology at study enrollment, n (%)**		
*P*. *aeruginosa* only		10 (56)
*P*. *aeruginosa* and *S*. *aureus*		7 (39)
*Pseudomonas* and other (*A*.*baumanii*, *A*. *xylosoxidans*, *E*. *coli*)		3 (17)
**Bacteria density** ^**a**^ **(CFU/mL)**		
Total Bacterial density- Pre-treatment ^b^		8.8 E7 ± 9.9E7
Total Bacterial density- Post-treatment		6.0E7 ± 1.4E8
*P*. *aeruginosa* pre-treatment		8.6E7 ± 1.1E8
*P*. *aeruginosa* post-treatment		5.9E7 ± 1.5E8
*S*. *aureus* pre-treatment		4.0E7 ± 4.6E7
*S*. *aureus* post-treatment		2.0E7 ± 3.4E7

^a^ All values are depicted as means ± SD, unless otherwise noted.

^b^ p< 0.05 for pre vs post comparison, paired t-test.

^c^ p<0.05 for pre vs post comparison on untransformed data using Wilcoxon signed rank test.

Overall, at the time of the study, subjects’ pulmonary functions were representative of CF adults in the US age 18 years or older, with a median percent predicted FEV_1_ (ppFEV_1_) of 57% prior to treatment with AZLI [20]. Half the subjects harbored chronic respiratory infection solely with *P*. *aeruginosa*; whereas, the other half additionally grew other pathogens, including *S*. *aureus*. The mean age of the cohort was 32 years, with a 44% male distribution. Consistent with the prevalence of F508del in the U.S. Caucasian population, 50% of subjects were homozygous for the F508 deletion.

**Fig 2:**
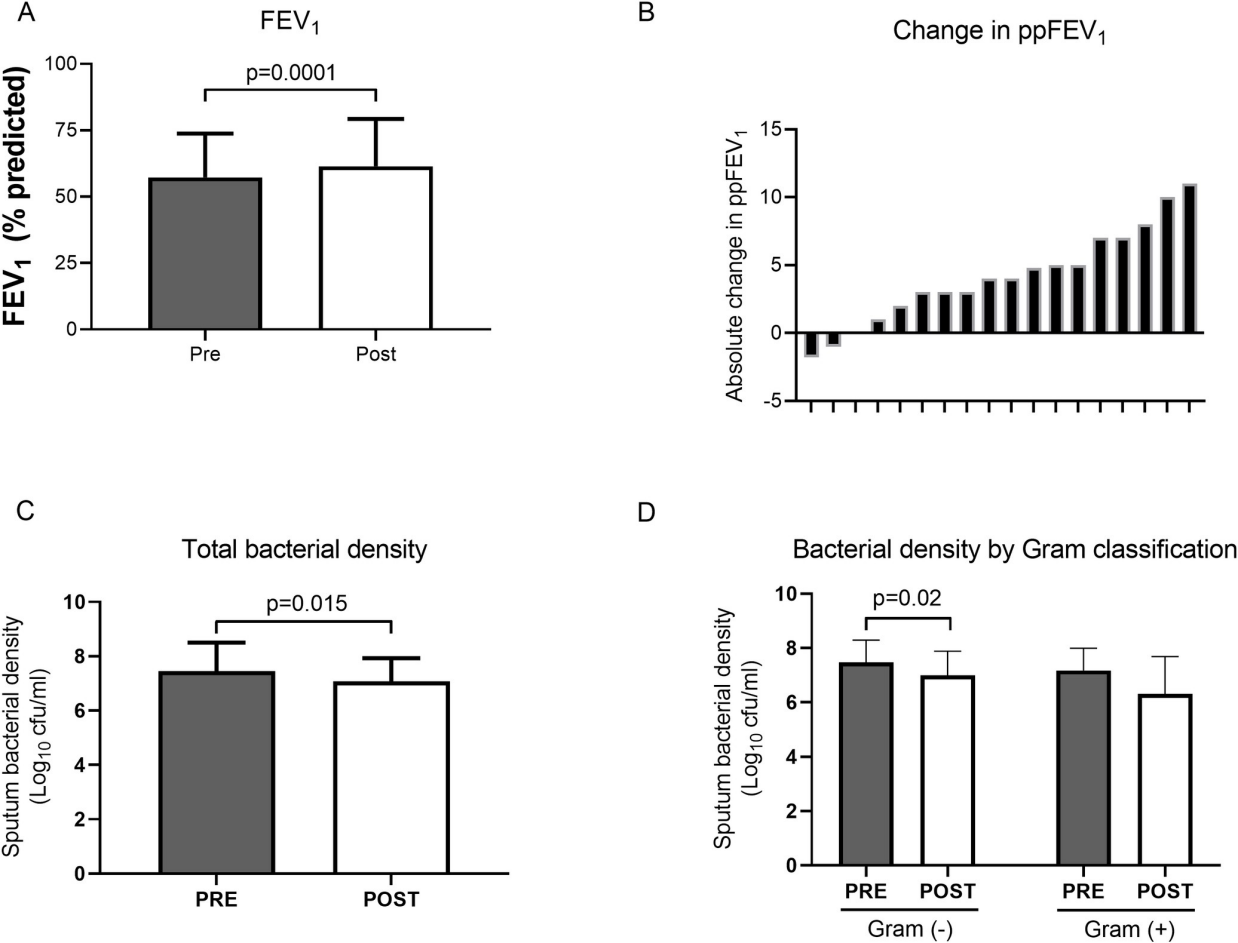
Effect of inhaled AZLI on standard variables pre and post treatment. (A) percent predicted Forced Expiratory Volume in the first second (ppFEV_1_), (p = 0.0001, paired t-test); (B) Waterfall plot demonstrating ppFEV_1_ change per patient, each subject represented by tick marks on x-axis; (C) Total bacterial density based on quantitative sputum cultures (p = 0.015, paired t-test following log transformation); and (D) bacterial density by gram staining classification. Gram negative organisms showed a significant decrease post treatment (p = 0.02, paired t-test following log transformation). Solid columns represent pre-treatment; open columns represent post-treatment. Bars represent mean values and error bars represent SDs on the same scale as means.

As a group, subjects demonstrated small but significant absolute improvements in their ppFEV_1_, with mean values rising from 57% to 61% (p = 0.0001), following one month of AZLI treatment (Fig 2A). Responses in ppFEV_1_ showed marked variation, as seen by waterfall plot (Fig 2B). Treatment resulted in significant decreases in total sputum bacterial density, from mean 7.6 to 7.1 log (10) CFU/mL (p = 0.015), as shown in Fig 2C. When microbiology results were partitioned into gram negative and gram positive pathogens (Fig 2D), gram negative pathogens declined from mean 7.3 to 7.0 log (10) CFU/mL (p = 0.02). There was no significant mean decrease in gram positive bacterial density following treatment. While *Pseudomonas* counts trended downward, the decline in burden did not reach statistical significance. Respiratory symptoms, assessed by the Respiratory Symptoms Scale of the Cystic Fibrosis Questionnaire-Revised (CFQR-Respiratory), were not significantly different following treatment.

**Fig 3:**
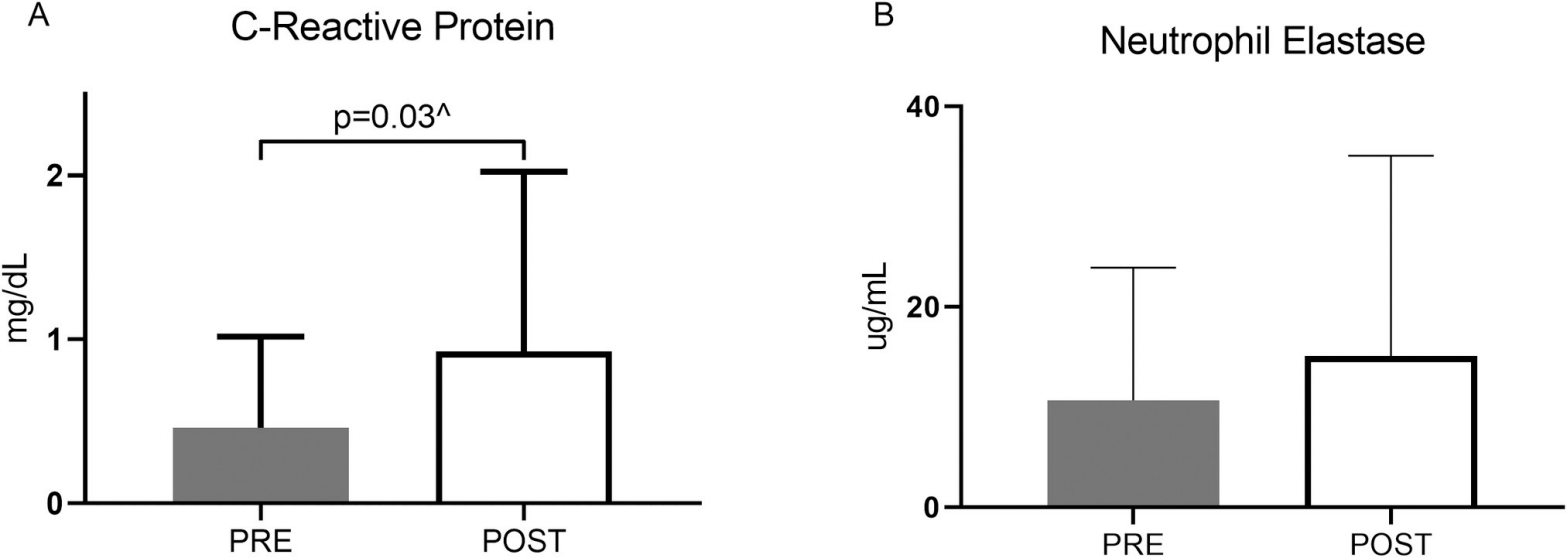
Effect of inhaled AZLI on markers of inflammation pre and post treatment. (A) C-reactive protein (CRP) measured from blood samples. ^p = 0.03 for Wilcoxon matched-pairs signed rank test. (B) Sputum neutrophil elastase. Comparisons were based on paired t-tests, from log-transformed data. Solid columns represent pre-treatment; open columns represent post-treatment. Bars represent mean values and error bars represent SDs on the same scale as means.

Markers of local sputum inflammation in addition to systemic inflammatory markers were evaluated in the cohort. In Fig 3A, interestingly, standard marker of inflammation, C-reactive protein (CRP), increased following treatment, from mean 0.48 to 0.92 mg/dL (p = 0.07 for paired t-test on log-transformed data; p = 0.03 for Wilcoxon signed rank test on untransformed data). Sputum neutrophil elastase in Fig 3B, trended higher at the post treatment visit, though the increase was not statistically significant.

**Fig 4:**
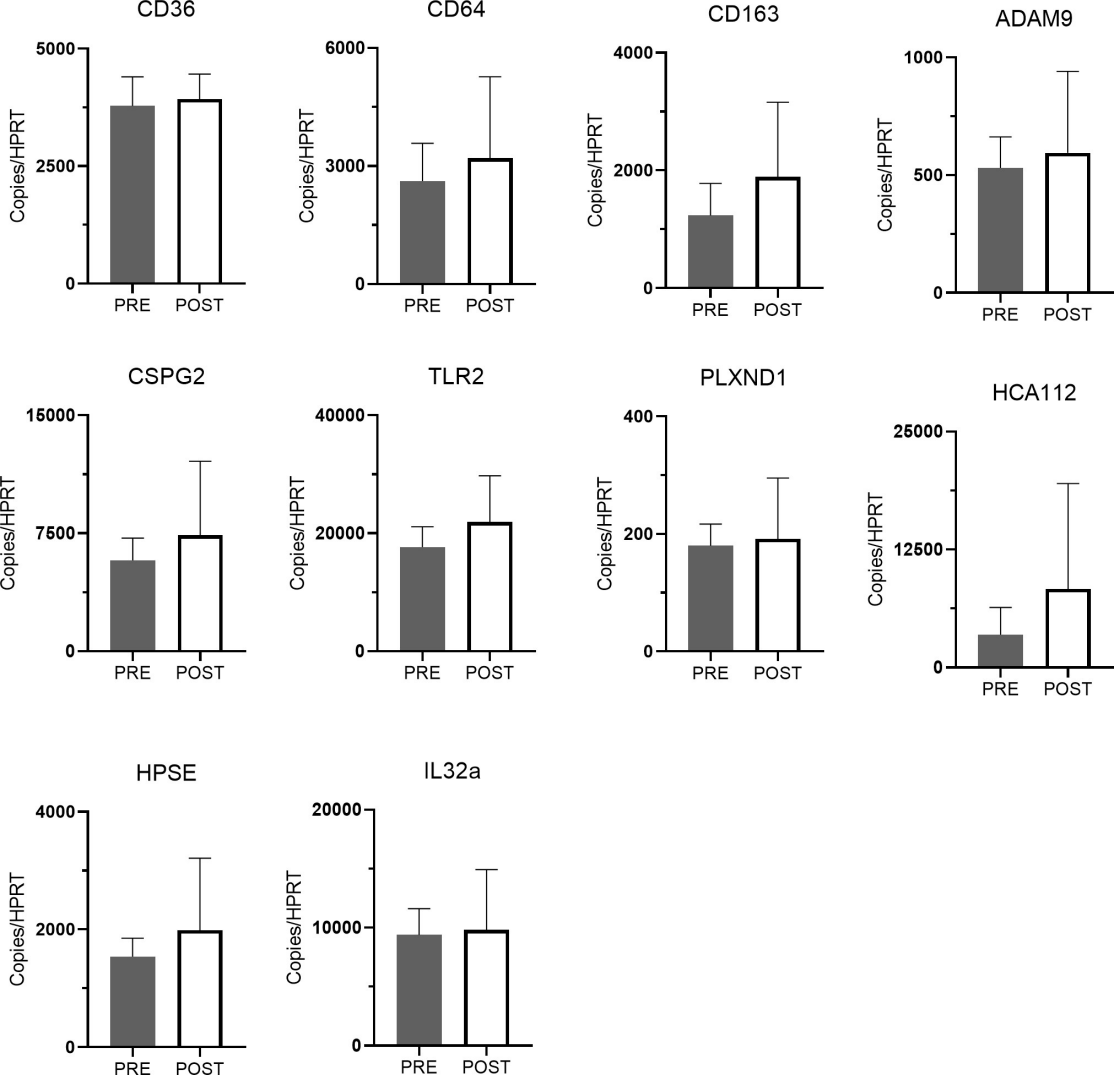
Effect of inhaled AZLI on mean leukocyte gene expression from whole blood pre and post treatment. Solid columns represent pre-treatment; open columns represent post-treatment. Bars represent means (± SD) relative to the detection of housekeeping gene *HPRT*. Differences analyzed by paired t-test following log transformation.

In terms of changes in whole blood gene expression, no single gene from the 10-gene panel manifested statistically significant change in expression following treatment (Fig 4). However, a multivariable linear mixed model for FEV_1_ chosen with backward selection included genotype (P = 0.08), *CD64* (p = 0.05), *CSPG2* (p = 0.02), *HCA112* (p = 0.005) and time (pre to post; p = 0.0007). In this model, the greater the expression of *CD64* and *CSPG2*, the lower the mean ppFEV_1_, whereas the greater the expression of *HCA112*, the higher the mean ppFEV_1_. Average ppFEV_1_ improved from pre to post time points in models with and without gene expression variables (p<0.001 for the time effect in both models). The addition of the 3 gene expression variables into the model improved prediction of ppFEV_1_ (p = 0.0001 for chi-square test comparing -2 log likelihoods for nested models; gene expression variables modeled on natural log scale).

**Fig 5:**
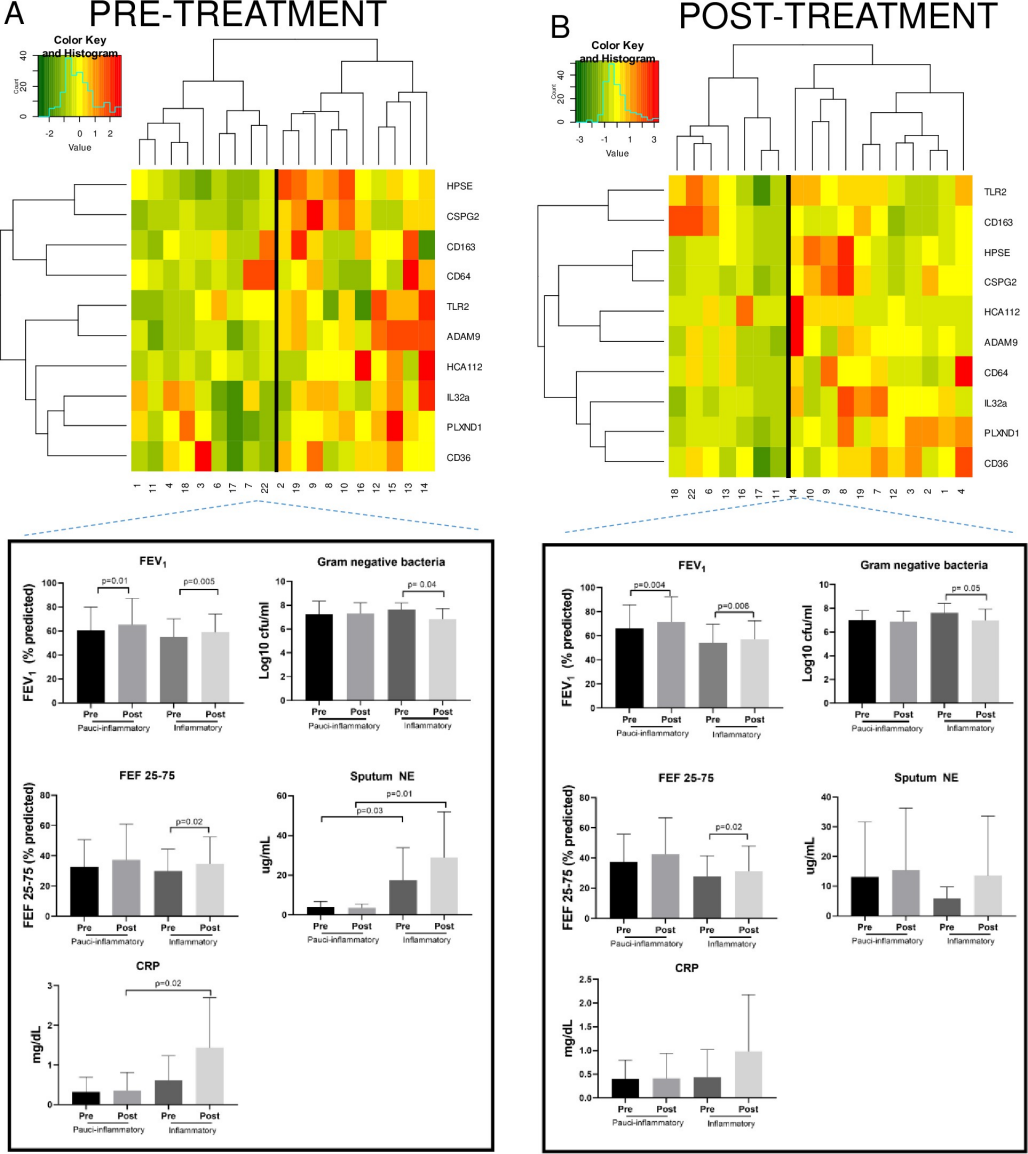
Dendrogram, heat map, and clinical characteristics for hierarchical clustering based on panel expression pre and post treatment. (A) The pre-treatment dendrogram only includes gene expression values prior to AZLI treatment and (B) the post-treatment dendrogram only includes gene expression values following AZLI treatment. Each column represents an individual patient and each row represents a single gene. For each dendrogram, subject IDs are depicted below each column, so that changes from pre- to post-treatment endpoints for each subject can be visualized. Expression of genes for each subject is depicted with a color-coded histogram representing level of gene expression on a natural log scale. Green signifies reduced gene expression; red indicates increased expression. Clusters are labeled “Pauci-inflammatory” or “Inflammatory” based on the degree of gene expression for pre and post-AZLI time points. The clinical characteristics of subjects in each cluster before and after inhaled AZLI are represented in the figures below each dendrogram to include ppFEV_1_, ppFEF_25-75_, gram negative bacterial density (log_10_ CFU/mL), sputum neutrophil elastase (μg/mL), each of which are evaluated by two sample t-tests. Bars represent mean values and error bars represent SDs on the same scale as means.

Hierarchical clustering of gene expression for the cohort was performed using two separate analyses, the first based solely on pre-treatment gene expression (Fig 5A) and the second based only on post-treatment gene expression values (Fig 5B). Both analyses identified 2 molecular clusters, delineated by the heatmaps in Fig 5. Expression data for the 10-gene panel is shown such that green shading indicates reduced expression and red indicates increased expression. Based on overall expression patterns, Group 1 was entitled “Pauci-inflammatory” and Group 2, which generally depicted greater transcript abundance of the panel, was labeled “Inflammatory”. Among the subjects whose pre-treatment genes clustered into the Pauci-inflammatory pre-treatment group, 56% remained in that group post-treatment. This is in comparison to the 70% of subjects whose pre-treatment genes clustered into the Inflammatory group and remained in that group post-treatment. We evaluated differences in clinical characteristics between the two clusters based on gene expression pre-treatment and post-treatment.

In order to identify whether the 10-gene panel could delineate subjects with baseline differences in inflammation prior to the start of therapy, we evaluated the relationship of pre-treatment gene expression with clinical response and additional measures of inflammation. This evaluation was done by comparing changes within and between clusters. As seen in Fig 5A, representing pre-treatment gene expression, the Inflammatory cluster manifested significantly greater sputum neutrophil elastase at treatment onset and treatment completion, compared to the Pauci-inflammatory group. This Inflammatory cluster also demonstrated significantly higher CRP at treatment completion (mean 1.43 versus 0.35 mg/dL in the Pauci-inflammatory group). Importantly, the Inflammatory group, but not the Pauci-inflammatory group, had significant reductions in gram negative bacteria following AZLI, with a decline from mean 7.62 to 6.82 log (10) CFU/mL post treatment, despite no differences in the pre-treatment burden (p = 0.04). Coinfection status with *S*. *aureus* was evaluated as a potential basis for increased inflammatory gene expression. *S*. *aureus* bacterial density was actually higher in the Pauci-inflammatory cluster than in the Inflammatory cluster at the onset of treatment, with no differences by treatment completion. When spirometry endpoints were compared between the Inflammatory and Pauci-inflammatory groups, both experienced significant improvements in ppFEV_1_. However, in evaluating small to medium airways, only the Inflammatory group manifested significant improvements in ppFEF_25-75_ (p = 0.02).

**Fig 6:**
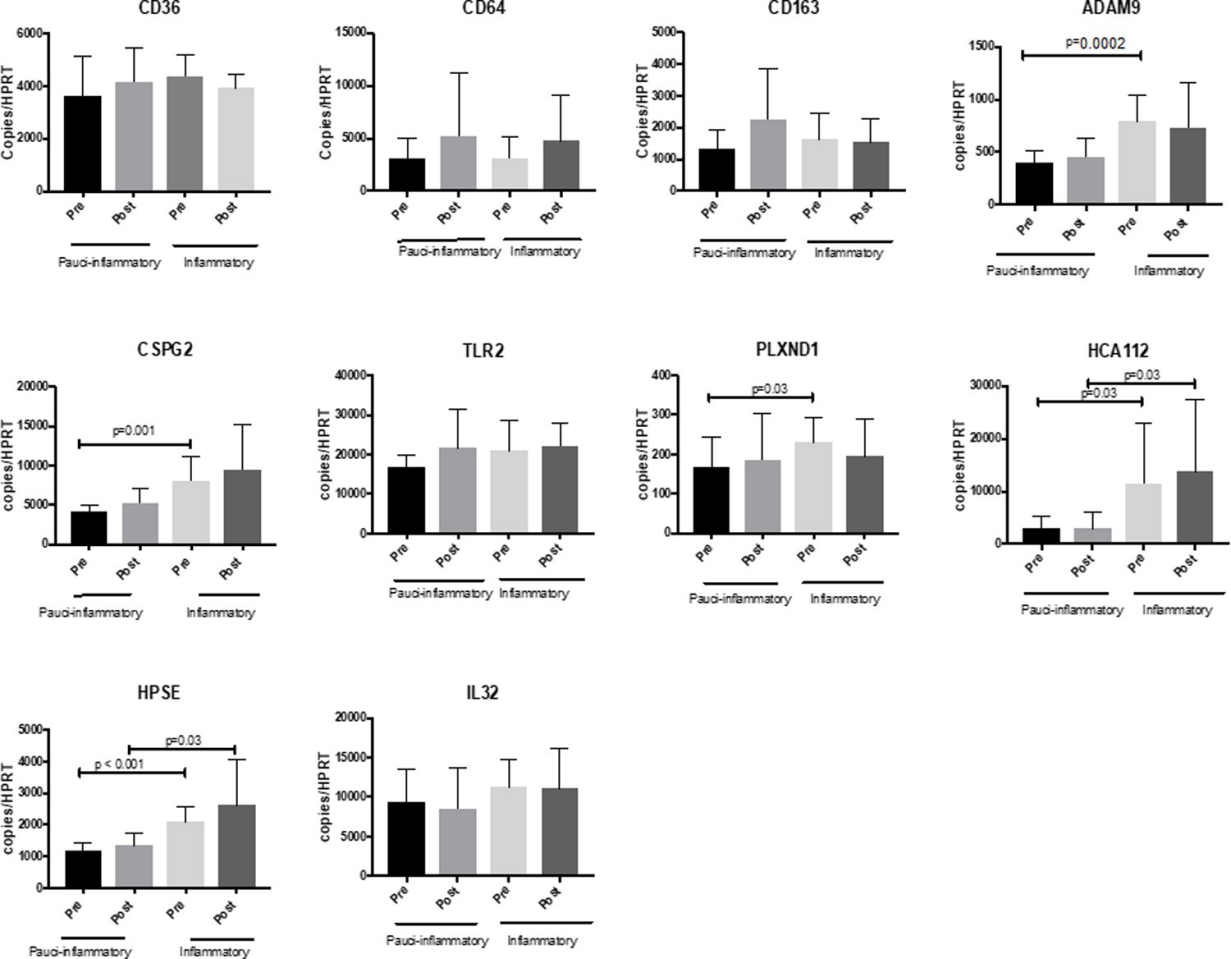
Whole blood gene expression stratifies AZLI treatment groups before treatment initiation, based upon hierarchical clustering. Pauci-inflammatory and inflammatory clusters are classified based on Pre-treatment dendrogram as shown in Fig 5, with expression of panel genes for each group. Again, columns depict means (± SD) relative to the detection of housekeeping gene *HPRT* and differences analyzed by paired t-test and 2 sample t-test following log transformation.

Given that the Inflammatory cluster manifested greater systemic and airway inflammation based on CRP and sputum NE, we evaluated whether specific genes within the panel were also representative of the group’s more inflamed phenotype, as seen in Fig 6. Consistent with sputum and systemic inflammatory variables, 5 genes (*PLXND1*, *ADAM9*, *CSPG2*, *HCA112*, and *HPSE*) were more highly expressed in the Inflammatory cluster at onset of treatment, while two of those genes (*HCA112* and *HPSE)* were also highly expressed in the Inflammatory cluster at post-AZLI time points.

In comparison, in the analysis of post-treatment molecular clusters (Fig 5B), NE and CRP values were not significantly different between the two groups. Both Pauci-inflammatory and Inflammatory clusters demonstrated significant increases in ppFEV_1_ and the Inflammatory group demonstrated significant increases in ppFEF_25-75_. The Inflammatory group demonstrated significant reduction in gram negative bacterial burden following AZLI.

## Discussion

While we have previously demonstrated that measurement of the 10-gene panel enhanced the assessment of treatment response following systemic therapy for pulmonary exacerbations [14, 19, 21], this prospective study presents the first application of the panel to identify changes in inflammation following inhaled antibiotic therapy during baseline health in CF. Given that treatment is localized to the airways and not systemic, changes in circulating leukocyte gene expression is a relatively novel finding. In particular, changes in blood or sputum inflammatory markers are usually non-significant in clinical trials of inhaled antibiotics [22]. Consistent with prior studies, single genes do not change significantly following treatment. However, analysis of the multigene panel identifies the relationship of genes with FEV_1_ response. By utilizing a panel, instead of single genes, multiple inflammatory pathways are represented simultaneously, such that the orthogonal nature of the genes reflect various disease pathways and enhance the predictive value of the test [14]. In particular, the test may have utility in stratifying response groups, based upon underlying inflammation, prior to treatment.

Administration of AZLI in this small cohort led to significant reductions in bacterial density and improvement in FEV_1_, as seen in both AIRCF1 and AIRCF2 Phase 3 trials [9, 23]. Pre-treatment FEV_1_ percentages for the current cohort closely reflect values from the clinical trials (mean 54% and 55% predicted for AIRCF1 and AIRCF2 respectively), with ppFEV_1_ predicted ranging from 35–93% and mean and median values of 57% predicted at treatment onset. In AIRCF1, 38% of study participants enrolled with ppFEV_1_ <50% and 63% with ppFEV_1_>50%. Comparably, 28% and 72% of our cohort enrolled with ppFEV_1_ <50 and >50% respectively. As is commonly seen in populations with chronic use therapies, where effect declines over time, the mean ppFEV_1_ improvement in the current study more closely reflected a smaller percent change in the active comparator trial of AZLI versus TIS, across three 28-day courses [3]. Unexpectedly, all measures of inflammation rose following treatment with AZLI, notably sputum NE, plasma CRP, and the gene variables, which could reflect the host response to bacterial killing or an immunologic response to the drug itself. The increased measures of inflammation were not attributable to changes in clinical status or other medication change, based on the documentation by the examining physician at the time of the post-treatment study visit. Since inflammation increased with treatment in general, the utilization of a gene panel to predict treatment response to inhaled antibiotics posed a greater challenge.

Thus, as the multigene panel predicts inflammatory status under conditions of chronic infection, its greatest utility in the inhaled antibiotic setting may be to stratify differences in baseline inflammation, prior to treatment onset, which is less sensitive with current available standard measures such as FEV_1_ or CRP. In order to address whether subject sub-populations could be defined by gene expression, hierarchical clustering based on gene expression was undertaken and identified two distinct patient populations both at the onset and at the completion of treatment. The pre-treatment clustering algorithm of gene expression identified Pauci-inflammatory and Inflammatory clusters, both of which demonstrated improvement in ppFEV_1_. There were notable clinical differences between the clusters, with the inflammatory cluster showing higher NE at pre and post treatment and a higher post-treatment CRP. The Inflammatory cluster also manifested significant reductions in gram negative bacterial counts and significant improvements in distal airflows. Thus, this cluster represents a distinctive host phenotype, and the gene variables may serve as a surrogate marker for greater host-pathogen engagement and/or a more active status of chronic *Pseudomonas* pulmonary infection at the time of inhaled antibiotic administration, based on a heightened host immune response. The post-treatment clustering also demonstrated significant ppFEV_1_ for both clusters and distal airflow improvement for the Inflammatory cluster. However, clinical measures of inflammation were less distinct between groups, such that no significant differences were seen in sputum NE and CRP. In the post-treatment period, cluster membership appears to be more heterogeneous when stratifying based on clinical characteristics than pre-treatment. This phenomenon most likely occurs because following treatment, the inflammatory response may be two-fold, either secondary to the antibiotic effect on sputum bacteria or a reaction to the inhaled antibiotic itself, which may explain why CRP and NE rose simultaneously with FEV_1_.

As mentioned above, the panel’s genes comprise multiple inflammatory pathways simultaneously, such that their uncorrelated nature boosts the test’s predictive value. The genes most highly associated with FEV_1_ response in the regression analysis are expressed by multiple cell types and provide insight into various parallel immunologic processes which may be active at the time of treatment. Associated with FEV_1_ improvement, *HCA112*, or TMEM176A, encodes an acid-sensitive nonselective cation channel expressed in T cells of the Th17 lineage, Th17CD4+, γδT17, NKT17, as well as type 3 innate lymphoid cells (ILC3) [24–27]. In mouse models, its deletion impairs MHC II presentation of exogenous antigens by dendritic cells for priming of naïve CD4+ T cells and antigen specific proliferation of CD4+ T cells [28]. Associated with FEV_1_ decline, *CSPG2* (VCAN), which predicted FEV_1_ response, is an extracellular matrix proteoglycan, expressed by monocytes and lymphocytes. Its upregulation by leukocytes enhances their adhesion, activation, and accumulation within tissues [29]. The association of gene expression to treatment within the airway indicates that immune responses to inhaled antibiotics can be detected within the peripheral blood. Indeed, the increases in CRP following treatment supports the finding that systemic changes in inflammation are identifiable and quantifiable, even in the case of inhaled antibiotic therapy.

Incorporating validated panels of immune response into analysis of patient response may be valuable in phenotyping patients prior to treatment. A goal of future and larger studies would be to validate whether inflammatory differences define phenotypes of responders to AZLI and to inhaled antibiotics in general, given all the challenges which will be present in future studies of antimicrobial agents in the setting of CFTR modulating agents.

Several limitations of the study are acknowledged. The trial was conducted at a single center, with a small sample size, reducing power to identify significant changes in transcript abundance. Furthermore, blood mRNA biomarkers may be less sensitive in identifying local changes in airway inflammation in subjects at baseline health than they are in identifying the systemic changes in inflammation in subjects with pulmonary exacerbations. Subjects demonstrated relatively small ppFEV_1_ responses with AZLI, which also may have dampened changes in the expression panel. Finally, none of the study subjects were undergoing simultaneous treatment with CFTR modulators, thus the effect of modulator therapy on gene predictors and inhaled antibiotic response is unknown. Given that highly effective modulator therapy is approved for 90% of the CF population in the US, current ongoing studies are just beginning to identify secondary changes in sputum bacterial load and inflammation (NCT04038047, in addition to the RCOVER trial in the UK). Nonetheless, inhaled antibiotics will continue to be an important mainstay in the CF therapeutic regimen, given that most adult subjects chronically harbor respiratory pathogens and that CFTR correction by modulator therapy is partial. In the coming years, it will be important to identify how CF subjects respond to inhaled antibiotic therapies, particularly if FEV_1_ becomes a less sensitive endpoint and patients are less likely to produce sputum, which will markedly impact research trial design and results [30]. In the clinical realm, the choice of which inhaled antibiotic to manage chronic infection on an individualized basis will continue to be an important determination in the course of treatment decision making.

In conclusion, whole blood gene leukocyte expression identifies distinct populations of responders to inhaled antibiotic therapy prior to treatment. However, the small population size limits definitive conclusions. Future studies are needed to identify whether it may serve as an inexpensive additive tool to define baseline inflammation, responses to inhaled antibiotic therapy, and/or longitudinal progression of infection related to the disease. Systemic markers of inflammation may give insight into pleiotropic effects of inhaled antibiotics within the CF airway and the heterogeneity of their effects between subjects, ideally allowing for greater personalization of treatment regimens.

## Supporting information

S1 Checklist CONSORT 2010 checklist of information to include when reporting a randomised trial*. (PDF)

S1 File (PDF)
