# Supplementary material for: Blood mRNA biomarkers distinguish variable systemic and sputum inflammation at treatment initiation of inhaled antibiotics in cystic fibrosis: A prospective non-randomized trial
Source: PLoS One. 2022 May 5;17(5):e0267592. doi: 10.1371/journal.pone.0267592 (PMC9070878; doi:10.1371/journal.pone.0267592)
Supplement: S1 File — (PDF) [file pone.0267592.s002.pdf]

Protocol

Title: Ultrasensitive detection of reductions in CF airway inflammation following one month of inhaled Cayston®

Principal Investigator: **Milene Saavedra, M.D.**
Co-Investigator: **Jerry Nick, M.D.**

National Jewish Health IRB
APPROVAL
Date 07.30.2015
Signed dnc

Date: July 23 2015

TABLE OF CONTENTS

Page

PROTOCOL SYNOPSIS 3
1. BACKGROUND 5
2. OBJECTIVES 10
    2.1 PRIMARY OBJECTIVE 10
    2.2 Primary Hypothesis 10
    2.3. Secondary Hypotheses 10
3. STUDY DESIGN 11
    3.1 Study description 11
    3.2 Outcome Measures 12
    3.3 Study Risks and Safety Plan 12
    3.4 Benefits 13
4. MATERIALS AND METHODS 13
    4.1 Subjects 13
        Subject Recruitment and Selection 13
        Inclusion Criteria 13
        Exclusion Criteria 14
    4.2. Enrollment and Study Initiation Time Window 14
    4.3 Treatment with Cayston® 14
    4.4 Informed Consent 14
    4.5 Minorities and Women 14
    4.6 Study Assessments 15
    4.6 Laboratory Assay Methods 15
    4.7 Statistical Methods 16

REFERENCES 18

PROTOCOL SYNOPSIS

Title: Ultrasensitive detection of reductions in CF airway inflammation following one month of inhaled Cayston®

Objectives:

1. Prospectively validate changes in a 10-gene panel of circulating leukocyte transcripts before and after 4 weeks of Cayston® in an adult CF cohort.

Hypotheses:

Central Hypothesis: Changes in the expression of a panel of 10 circulating blood leukocyte genes can sensitively and reproducibly diagnose cellular and structural changes in pulmonary inflammation following Cayston® therapy.

Secondary Hypotheses

1. Sensitivity and specificity of transcriptional profiles exceed sensitivity and specificity of standard measures of FEV_1, CRP, CBC, serum IL-8, sputum IL-8, and neutrophil elastase patient reported outcomes and change in bacterial density.
2. Profiles predict Cayston® responders versus non-responders and predict which inflammatory pathways are most highly correlated with treatment response.

Study Design:

An investigator-initiated observational study at a single site.

1. Adult patients will be enrolled at the start of a month of inhaled Cayston® therapy, based on FDA criteria for use of the drug: as inhaled antibiotic prophylaxis for patients chronically infected with *Pseudomonas aeruginosa*, with FEV_1 of 25% or greater. Enrollment into this study will only occur once a patient's primary MD has made the decision to start this medication. Patients will not be started on this medication for the purposes of this study.
2. A 15cc sample of whole blood will be obtained within 24 hours of initiating a month long course of Cayston.
3. Patient demographics, medications, spirometry, sputum microbiology, CBC, and CRP will be recorded. A sample of expectorated sputum will be saved for IL-8 measurement. Serum IL-8 will be measured as well. Patients will complete a patient reported symptom scale, called the CF-Questionnaire-Revised (CFQ-R) Respiratory Scale.
4. Following 1 month of treatment, a second 15 cc sample of whole blood will be obtained +/- 3 days of completion of the therapy. Sputum microbiology, CRP, CBC, and spirometry will be repeated. A sample of expectorated sputum will be saved for IL-8 and neutrophil elastase quantification. Serum IL-8 will be measured as well.
5. A complete list of medications utilized during the month of therapy will be recorded.

Sample Size/Interim Monitoring:

Thirty-five patients will be enrolled, with the anticipation of a 15% dropout rate, in order to have 80% power to detect clinically significant changes in the expression of a single gene pre- and post- inhaled antibiotic therapy. The PI will monitor the protocol data on a quarterly basis for safety. This protocol does not pose significant risk, is studying the response to an FDA approved drug for CF treatment which is being prescribed clinically, and it is not blinded, randomized, or placebo-controlled. For these reasons, we anticipate that no DSMB will be required.

Inclusion Criteria:

1. Documented diagnosis of CF.
2. Age 18 years old or greater.
3. FEV_1 % predicted 25% or greater
4. Ability to perform reproducible Pulmonary Function Tests.
5. Willingness to comply with study procedure and willingness to provide written consent.
6. Clinically stable without evidence of acute upper or lower respiratory tract infection or current pulmonary exacerbation within the 14 days prior to the screening
7. Ability to produce at least 1mL of sputum spontaneously, or be willing to undergo sputum induction
8. Chronic bacterial colonization with Pseudomonas aeruginosa (2 documented positive cultures in the prior 2 years.)
9. Patient is starting on a cycle of Cayston as part of their clinical care.

Exclusion Criteria:

1. Presence of a condition or abnormality that, in the opinion of the Principal Investigator (PI), would compromise the safety of the patient or the quality of the data.
2. Aztreonam allergy, bronchospasm, or other contraindication to use of aztreonam.
3. Signs and symptoms of acute pulmonary exacerbation at the time of enrollment or during study.
4. Active infection and treatment for non-tuberculous mycobacteria.
5. Concomitant use of systemic steroids.
6. Use of inhaled antimicrobial agents with activity against *Pseudomonas aeruginosa* within 28 days prior to Visit 1.

1. Background

Airway infection and inflammation in CF

Lung disease in CF stems from dysfunction of the cystic fibrosis transmembrane conductance regulator (CFTR), resulting in dehydration and compression of the periciliary layer of fluid, impairing ciliary clearance [1]. Inflammation accompanies the CFTR defect, even in absence of detectable infection [2]. In CF lung disease, a continuous influx of neutrophils to the airway occurs from infancy, and even in young children, neutrophil accumulation to the airway lumen is disproportionably high compared to other inflammatory lung diseases [3]Faced with chronic inflammation, the capacity of the airway to clear the dying cells and cell products is overwhelmed. Clearly implicated in the destruction of the CF lung are proteases, especially elastase [4], and oxidant injury [5]. The collapse of the mucus layer and subsequent mucostasis is followed by characteristic infections, particularly *Pseudomonas aeruginosa*. Neutrophil-derived actin and DNA increase the tenacity of the sputum, and may enhance the formation of *P. aeruginosa* biofilms [6], which clearly accelerates CF lung inflammation. Anti-inflammatory treatments are of benefit in CF [7], but to date, antimicrobial therapy is the most effective method of acutely slowing the "vicious cycle", as reducing the bacterial burden indirectly results in a decrease in CF airway inflammation [8].

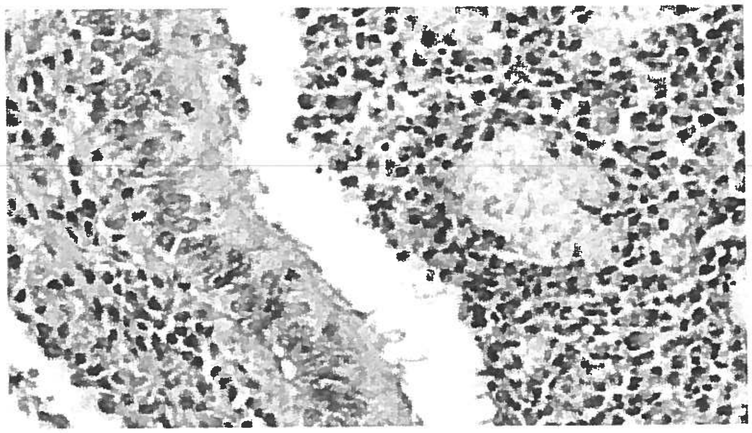

Figure 1. Section through a cartilaginous airway from a 7 y.o. female from The Children's Hospital (Denver) who succumbed to CF lung disease. The airway lumen contains demonstrates an intense neutrophil infiltrate. At higher magnification the submucosal infiltrate is seen to consist of lymphocytes. Monocytes are seen near areas of cartilaginous damage.

Lymphocytes and monocytes participate in CF airway injury

Although neutrophils and airway epithelial cells are the recognized mediators of inflammation in the lumen of CF airways, there is increasing evidence that mononuclear cells (lymphocytes and monocytes) participate in CF lung disease as well [9, 10]. T lymphocytes are the most numerous leukocyte population present in the CF bronchial wall and likely contribute to neutrophil trafficking [10]. Mononuclear cells are the predominant cell type found in areas of cartilaginous injury that is very characteristic of CF [9] (**Figure 1**). Mononuclear cells circulating in the peripheral blood (PBMCs) are the source for lymphocytes and monocytes involved in CF airways disease and hence may also provide clues to pathogenesis, particularly with respect to submucosal and cartilaginous inflammation and injury [9].

Pulmonary function testing is an inadequate outcome measures in CF

The established outcome measure in CF is FEV_1, a measure of airflow limitation. Historically, FEV_1 correlates with disease progression in CF [11] and is used routinely for both clinical care and to test the efficacy of new therapies. However, FEV_1 is an imperfect outcome measure for a variety of reasons:

- Decline in FEV_1 is nonspecific. Airflow limitation due to structural changes cannot be distinguished from airflow limitation due to mucus plugging, bronchoconstriction, or airway edema.
- Fibrosis and remodeling of the airway and lung parenchyma occur slowly, and potential benefits of anti-inflammatory treatments may not be apparent for months.

- Infants, young children, and severely ill adults may be unable to perform PFTs.
- FEV_1 is effort dependent, and can be reduced by a number of factors independent of airway obstruction.
- Newly available therapies have increased average FEV_1 and dramatically reduced the historical rate of decline, thus patients with early (mild) disease and normal FEV_1 may be unable to achieve a significant improvement in FEV_1, regardless of benefit by a particular therapy[12].

Potential impact of a sensitive biomarker in the clinical care of CF patients
The availability of a sensitive surrogate for lung inflammation would impact all aspects of CF care. Aggressive treatment of CF would likely benefit young children if biomarkers were available to identify those with more aggressive inflammatory disease and to evaluate treatment outcomes. Conversely, in patients with severe lung destruction, who are infected with multiple organisms, assessment of response to a particular treatment is often difficult, given day-to-day disease variability and irreversible airway damage. A diagnostic could be used to gauge the response to therapeutics in settings where the clinical response lags far behind. In addition, increasing numbers of therapeutic options will be available to CF patients in the future, but there is no guidance as to how various treatment options can be combined. The availability of a biomarker(s) could allow physicians and patients to personalize their care by being able to rapidly detect improvements (or lack there of) in airway inflammation with the addition (or subtraction) of various treatments. The implementation of clinical trials for CF is hampered by a lack of sensitive measures of treatment response. For patients on current recommended therapies, large improvement in FEV_1 is no longer achievable and frequently not demonstrable in two populations who have the most to gain from newly developed therapies: 1) pediatric populations unable to perform PFTs, and 2) patients with very mild disease whose PFTs do not significantly change in response to treatments.

Potential impact of a sensitive biomarker in CF clinical research
Although a multitude of candidate therapies are being proposed for the treatment of CF, few are being made available to the patient [13]. While 453 clinical studies of CF therapies were identified by PubMed in 2006 (compared to 19 publications in 1990), only three therapies have resulted in FDA approval for CF lung disease [13]. Airway inflammation is also a valuable secondary endpoint for other categories of drugs under development, as improvements in antibiotics, mucous treatment, protein repair, salt transport and nutrition could all be expected to contribute to decreased airway inflammation. As a multitude of strategies are emerging, application of these therapies to CF threatens to lag far behind. The conduct of definitive phase 3 trials requiring more than 500 patients often requires multinational participation, adding significant complexity and cost [13]. More importantly, pivotal phase 2 trials can decide against a potentially useful therapy if they are underpowered. The capacity of the CF Therapeutic Development Network (TDN) to study these potential treatments in large clinical trials is severely limited, thus a more facile study design is needed that will allow rapid evaluation of anti-inflammatory treatments in CF with a minimum number of subjects. Finally, the validation of sensitive surrogate markers is a particular mission of the FDA, though a key quest in identifying surrogates is an understanding of the biological relationship between the surrogate and the clinical outcome, in this case improved survival due to reduction of inflammation [14].

The following characteristics are required for a biomarker of CF airways inflammation:
- Sensitive indicator to reflect changes in clinical status.
- Rapid and inexpensive.
- Widely available in clinical settings, independent of specialized research centers.
- Elevated in the CF population during periods of baseline health, so response to chronic therapies can be assessed during the stable state.

- Biologic relevance to CF lung disease.

Biomarkers found in circulation have many advantages in monitoring lung disease.
While the inflammatory changes of CF are of greatest intensity in the airways, there are many factors that have limited the development of biomarkers sampled directly from the airway. Although inflammatory changes in the circulation are far less pronounced than those in the airway, monitoring this compartment has obvious advantages, including:

- Samples are easily obtained, independent of patient effort or specialized techniques.
- Blood passes through the lung continuously, thus rapidly reflects changes in inflammation.
- Blood passes through all lobes, resulting in a "summation" of the total pulmonary inflammation and reducing potential sampling variability from very heterogenic disease distribution.
- Blood leukocytes are the source of inflammatory cells in the lung, thus are directly relevant to the pathophysiology of CF inflammation.

Cayston®, which is inhaled aztreonam, is a useful model of successful anti-infective and anti-inflammatory treatment in certain CF populations.
Cayston® was FDA approved in February 2010 as a chronic inhaled antibiotic therapy for CF patients colonized with *Pseudomonas aeruginosa* and an FEV1 >25% and <75% predicted. Phase 3 studies of the drug demonstrated improvements in the primary endpoint, change in patient-reported respiratory symptoms, as well as all secondary endpoints: change in pulmonary function, hospitalizations, use of non-study anti-pseudomonal antibiotics, change in the non-respiratory domains of the CFQ-R and missed school/work days. *P. aeruginosa* sputum density significantly decreased by -1.384 $\log_{10}$ CFUs by treatment end (Day 28) ($p \leq 0.001$). Mean FEV_1 increased 7.9% after month-long treatment ($p \leq 0.001$ vs. placebo). Use of non-study drug anti-pseudomonal antibiotics (Days 0 to 42) was significantly less for Cayston® treated patients (17.5%) than for placebo-treated patients (35.7%; $p = 0.013$). Despite these improvements, current standard measures of treatment response did not demonstrate a significant change in patients with an FEV_1>75% predicted. This may be in part due the known limitations in sensitivity of measurements such as FEV_1 for reductions in inflammation.

Preliminary studies

Peripheral Blood Mononuclear Cell (PBMC) gene expression signature before and after treatment for a CF pulmonary exacerbation.
PBMC gene expression has been used as a biomarker in a number of inflammatory lung diseases [15-17] and is very reproducible in the same individual [18]. Recently, we reported the PBMC "expression signature" in response to the treatment of a pulmonary exacerbation in CF [19]. PBMCs were isolated before and after treatment, RNA was extracted, and genomic analysis was conducted according to a standard algorithm. Beginning with microarrays containing over 35,000 sequences from 10 patients before and after antibiotic treatment, we focused on sequences assigned to known (named) genes with a minimum expression threshold (Affymetrix "Present call"). The study design utilized "within subject comparisons", such that each study subject served as their own control. From these genes, we identified differentially expressed genes in pairwise comparisons between the pre-treatment and post-treatment groups, using the non-parametric MannWhitney U test, with a minimum of 1.4-fold change. This analysis yielded 32 candidate genes with both significant expression and significant change. Screening for **biologic plausibility** and confirmation by real time PCR reduced the list to 10 genes (**Table 1**), all of which will be tested in this proposal. While 9 of 10 genes decreased in expression with treatment, IL-32 increased with treatment, reflecting the specificity of the assay. Of interest, when compared to PBMC isolated from age and sex-matched normal subjects, pre-treatment expression of 9 out of 10 CF candidate genes was significantly different from normal subjects,

while following treatment only 1 of 10 was significantly different from normal subjects. In every case, following antibiotic treatment, CF gene expression more closely resembled the healthy age and sex-matched normal subjects [19]. Finally, the entire gene signature was validated in an independent patient cohort (n=14) [19].

Genes in the CF therapeutic signature are linked to inflammation and host defense

Genes identified in the CF therapeutic signature were screened for biological plausibility [19]. Seven of the 10 genes and their products (IL32, HPSE, ADAM9, PLXND1, HCA112, CSPG2, and CD163) have not previously been linked to CF lung disease, but are implicated in pathologic pulmonary inflammation, manifested in asthma and pneumonia [20, 21]. The fact that these genes encode for proteins implicated in inflammatory processes, lends biologic plausibility to naming them as potential markers of resolution of CF airway infection and inflammation. As a group, these genes represent functions of immune recognition and response, phagocytosis, and matrix degradation. Toll-like Receptor 2 (TLR2) is a central pattern recognition receptor for the innate response against bacterial infection. IL-32 is a newly described TNF-inducible intracellular cytokine which induces blood monocyte differentiation to macrophages, with subsequent phagocytic activity for live bacteria [22, 23]. Three surface receptor genes participate in phagocytosis. CD64, or FcγRIA, mediates receptor mediated endocytosis of IgG-antigen complexes in macrophages [24]. CD36, a scavenger receptor, mediates macrophage uptake of oxidized LDL, as well as serving as a surface receptor for thrombospondin-1 [25-27]. CD163 serves as a macrophage cell surface hemoglobin scavenger receptor, and was recently shown to be highly predictive of mortality in pneumococcal bacteremia [20, 28]. Degradative enzymes, including heparanase, ADAM9 and versican, facilitate extravasation of leukocytes to inflamed tissues. In persistent airway inflammation, this process may culminate in marked and irreversible structural injury to lung parenchyma, by modification of extracellular matrix architecture [29].

Table 1: PBMC genes in the CF therapeutic gene signature

	Gene	Description	Fold change[1]	p values[2]	Expressed by leukocytes
Cell membrane molecules and receptors	CD64	Fcγ Receptor IA	-2.4	0.004	PMN, monos, mDC
	CD36	collagen type 1 receptor/thrombospondin receptor	-1.7	<0.001	CD4+ T cells, monos, DC
	CD163	hemoglobin scavenger receptor	-1.6	0.003	Monos, PMN's
	TLR2	toll-like receptor 2	-1.6	0.003	CD4+ T cells, monos, PMN's, B cells, DC
	HCA112	hepatocellular carcinoma-associated antigen 112	-1.8	0.004	lymphocytes, monos, DC
	PLXND1	plexin D1	-1.7	0.005	Monos, fDCs
Immune response	IL32	interleukin 32	1.4	0.033	Activated T cells, NK cells, monos
Matrix degradation/ Extravasation	HPSE	heparanase	-1.4	0.017	Monos, PMNs
	ADAM9	a disintegrin & metalloproteinase, meltrin gamma	-2.5	0.004	CD4+ T cells, monos
	CSPG2	versican, chondroitin sulfate proteoglycan 2	-1.9	<0.001	Monos

[1]Mean fold changes compare pre- and post-antibiotic expression values from oligonucleotide arrays

[2]p values calculated by paired t-test following log transformation

DC (dendritic cells, myeloid (m) and follicular (f)), NK cells (natural killer cells), PMN (neutrophils), monos (monocytes and macrophages)

Diagnostic value of the CF therapeutic signature in association with % change in FEV_1.

In a multivariate analysis, we evaluated the combined explanatory power of FEV_1 in combination with gene expression values for predicting response to therapy. In a logistic regression model, the genes reproducibly added independent predictive power to FEV_1, and represent a novel tool to quantify CF therapeutic response noninvasively. From the 10-gene signature, 7 genes were strong independent predictors for treatment response in the regression model for the 2 groups. Three genes significantly improved diagnostic value ($p \leq 0.05$) in both cohorts. ROC analyses reflected the overall diagnostic value of the gene markers, in terms of sensitivity and specificity compared to FEV_1 alone. The independent, significant explanatory power contributed by these genes demonstrates that gene expression

values from the CF therapeutic signature enhance the predictive discriminating value of FEV_1 alone[19]. All 10 genes encode for proteins implicated in inflammatory processes, lending biologic plausibility to their potential role as markers of resolution of CF airway infection and inflammation.

The CF gene signature may be assayed directly from whole blood
In the initial 2 cohorts of 24 CF patients presenting with acute exacerbations, a panel of circulating leukocyte genes in combination with FEV_1 significantly outperformed FEV_1 alone in predicting resolution of pulmonary inflammation[19]. However, measurements were labor intensive, requiring isolation of peripheral blood mononuclear cells (PBMCs) from whole blood. Until now, these results have never been tested in a larger, diverse CF cohort, nor has the assay been validated in a simpler format, such that quantification may be performed from whole blood. In 2007, a CFFT Award entitled "CF Leukocyte Genes as biomarkers for novel therapies" (P.I. Jerry Nick, ClinicalTrials.gov Identifier: NCT00727285) allowed validation of these findings in 60 subjects utilizing a whole blood platform, which obviates the need to isolate PBMCs prior to measuring the inflammatory gene panel. Gene expression of the CF gene signature before and after intensive antibiotic treatment was tested directly from whole blood RNA in a cohort of CF subjects presenting with acute exacerbation, isolated simultaneously with PBMC mRNA. The expression signature detected in PBMCs was mirrored in RNA transcripts from whole blood. The manuscript for this study has just been completed, and the data confirms that gene expression of this panel of circulating leukocytes, tested directly from whole blood, is a highly sensitive, specific and accurate marker of resolution of pulmonary infection and inflammation, following treatment of an acute exacerbation (Table 4 below). Incorporation of the whole blood gene panel with FEV_1 increased diagnostic accuracy for treatment effect from 64 to 81% and specificity for identifying those correctly predicted to have a reduction in inflammation from 54% to 77%, over FEV_1 alone. In multiple logistic regression analysis, six genes from the whole blood panel were significant predictors of response, beyond FEV_1 and CRP alone, adding diagnostic power to standard clinical variables.

Table 4: Multivariate analysis of mRNA transcripts and standard outcome variables for treatment effect in CF

VARIABLE	SENSITIVITY	SPECIFICITY	PPV	NPV	ACCURACY
FEV_1	74	54	62	67	64
CRP	84	53	64	76	68
PBMC panel	75	53	61	67	64
Whole blood panel (plus FEV_1) (ALL)*	84	77	79	83	81
Whole blood panel (plus FEV_1)**	80	86	85	81	83

*Values reflect analysis of entire cohort
**Values reflect analysis of subset which returned to baseline FEV_1 at treatment end

Diagnostic value of the CF therapeutic signature in combination with change in FEV_1 when evaluated from whole blood

ROC analysis revealed greater predictive accuracy when genes from the CF signature were combined with FEV_1 to diagnose resolution of airway inflammation. Using gene measurements from whole blood, a 7-gene panel with FEV_1 markedly outperforms FEV_1 alone (**Figure 2**). Using ROC curves to determine sensitivity and specificity, the combinations of genes plus FEV1 produced an area under the curve (AUC) of 0.85, versus an AUC of 0.63 for FEV1 alone. An AUC of 1 indicates a perfect test.

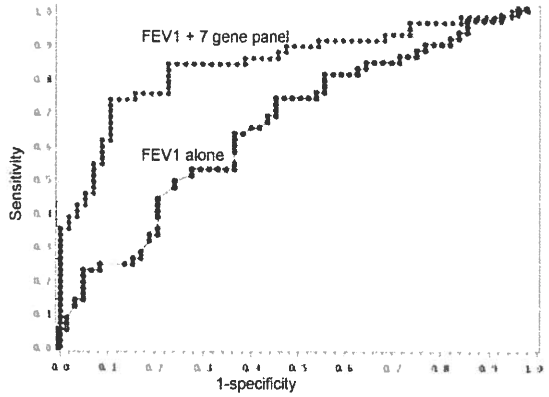

Figure 2: Juxtaposed ROC curves for diagnostic accuracy of FEV_1 alone (AUC= 0.63) versus FEV_1 + 7 gene panel (AUC=0.85).

Summary of current gaps in technology and knowledge

- We do not know to what extent changes in gene expression reflected in treatment of pulmonary exacerbations may be applicable to candidate therapies for treatment of CF lung disease until biomarkers are directly tested in the context of those therapies. FEV_1 and CRP, standard measures of therapeutic outcome, clearly underperform when used singly versus in combination with leukocyte transcripts.
- An assay of whole blood gene expression to test candidate CF therapies must be validated in a single-site trial before proceeding to multi-site testing. Furthermore, this should be done using a platform that is broadly available and simple to perform.
- Since FDA approval in 2010, Cayston has joined the armamentarium of standard treatments for CF lung disease. While Cayston is FDA approved as an inhaled anti-pseudomonal antibiotic in CF adult patients with FEV_1 25-75% predicted, FEV_1 benefit has not been shown in patients with FEV_1 > 75% predicted, nor has it been significant in pediatric populations. This is particularly due to lack of other sensitive outcomes measures by which treatment effect may be measured. More sensitive identifiers of treatment response may ultimately identify other CF populations who may benefit from this therapy.

2. Objectives

2.1 Primary Objective:

1. Prospectively validate changes in a 10-gene panel of circulating leukocyte transcripts before and after 4 weeks of Cayston® in an adult CF cohort.

2.2 Primary Hypothesis: Changes in the expression of a panel of 10 circulating leukocyte genes serve as a sensitive measure of treatment effect of month long Cayston® therapy.

2.3 Secondary Hypotheses

1. Sensitivity and specificity of transcriptional profiles exceed sensitivity and specificity of standard measures of FEV_1, CRP, CBC, the CF Health Related Quality of Life questionnaire, serum IL-8, sputum IL-8, and neutrophil elastase and change in bacterial density.

2. Profiles predict Cayston responders versus non-responders and also predict which inflammatory pathways are most highly correlated with treatment response.

3. Study Design

3.1 Description of the Study

CF patients enrolled in this proposal will be recruited from patients followed by the Adult CF Program at National Jewish Health. We project enrolling 35 subjects total, with CF ≥ 18 years of age when starting a month of prescribed Cayston® therapy while at baseline health. Patients will be selected based on planned use of Cayston® by their primary physician. <u>No patient will be started on Cayston® for the purpose of the study</u>. All subjects will have a baseline FEV_1 of 25% predicted or greater. Other standard aspects of chronic CF care, including inhaled mucolytics and azithromycin use will continue as prescribed. Concomitant use of systemic steroids will not be allowed, and co-infection with non-tuberculous mycobacteria will result in exclusion.

Following screening and enrollment, blood will be collected at two different time points. The first samples will be collected within 24 hours of starting inhaled Cayston® therapy. The second blood specimen will be collected at the end of the month long Cayston® treatment. At the time of each blood draw, CRP, CBC, a sputum sample for quantitative microbiology, sputum IL-8 and neutrophil elastase, and simple spirometry will be measured. A total of **15 mls of blood** will be required for this study at each draw.

Table 2: **Time-events schedule for study enrollment**

Measurement/Event	Day 0-1	Day 28 +/-3
Demographics (age, gender), history and physical	X	
CF diagnosis history (mutation)	X	
CF related conditions (CFRD, ABPA, Liver disease, exacerbations)	X	X
Medications in last 30 days	X	X
Inhaled and IV antibiotic therapy in the previous year	X	
Quantitative microbiology results	X	X
Serum IL-8	X	X
CRP and CBC	X	X
Sputum IL-8 and neutrophil elastase	X	X
Spirometry (FEV_1, FVC, FEV_1/FVC and FEF_{25-75})	X	X
CFQ-R	X	X
Whole blood for PCR analysis of gene expression	X	X

A schedule of study events is summarized in **Table 2**. At the time of enrollment and pre-treatment blood draw (Day 0-1), each subject will have a medical history, standard physical examination with the following diagnostic information obtained on all enrolled subjects and entered onto Case Report Forms (CRF): demographic information (age, gender, ethnicity), CF diagnostic history (sweat test results, CF genotype), history of CF-related medical conditions (CF-related diabetes, ABPA, CF-related liver disease), number of pulmonary exacerbations over the past year requiring antibiotics, hospitalizations in the preceding year, all medications being taken within the preceding 14 days, and microbiology results in the preceding year. In addition, all medications prescribed for the duration of the study, will be included on the patient's CRF. Patients must be at least 2 weeks out from treatment for a pulmonary exacerbation in order to be enrolled in the study. Enrolled subjects will also complete a CF-Questionnaire-Revised (CFQ-R) Respiratory Scale, a patient reported symptom scale, at the onset and completion of inhaled Cayston® therapy. The CFQ-R is designed to measure CF-specific patient reported health-related quality of life.

At the initiation of treatment (Days 0-1) and completion of treatment (Day 28 +/-3), blood will be collected for evaluation of leukocyte transcripts from whole blood, as proposed in this application. Sputum will be collected by spontaneous expectoration for bacterial identification, neutrophil elastase and IL-8 measurement, serum IL-8 quantified, and spirometry will be performed (FEV_1, FVC, FEV_1/FVC ratio, and FEF_{25-75}).

3.2 Outcome Measures

Primary Outcome Measure

The Primary analysis is the change in expression of individual and combinations of mononuclear cell genes, obtained pre- and post- Cayston® therapy.

Secondary Measures

Secondary outcome measures include FEV_1, CRP, CBC, CFQ-R, serum IL-8, sputum IL-8, and neutrophil elastase and change in bacterial density.

3.3 Study Risks and Safety Plan

Data and Safety Monitoring Plan for this Protocol: This protocol does not pose significant risk, is utilizing an FDA approved drug in the approved population, and it is not blinded, randomized, or placebo-controlled. Patients will not be started on drug for the purposes of the study, but will be recruited to the study once their clinic physicians have decided to prescribe inhaled Cayston® as part of their treatment regimen, or they are ready to start a cycle of their regular every-other month of Cayston. For these reasons, we anticipate that no DSMB will be required. The PI will monitor the protocol data on a quarterly basis.

Description of any Serious Adverse Events (SAE) That Are Possible for This Protocol: The procedures to be performed on the subjects in this protocol are essentially noninvasive forms of testing. Therefore, the risks associated with this study are believed to be minimal. Subjects may experience pain, discomfort and bruising with blood draws. Pulmonary function testing may theoretically induce mild shortness of breath or bronchospasm, but this has not historically resulted in our clinic. For the purpose of this protocol, an SAE will be bronchoconstriction resulting in a reduction in O_2 saturations below 85% or a significant drop in FEV_1 that does not respond to administration of albuterol or, requires monitoring and intervention for more than 30 minutes beyond the completion of the procedure.

Plan to Protect Subjects & Mitigate Risks: Subjects will be monitored closely for any adverse events. Blood draws will be performed by experienced research nurses at the Clinical Research Unit (National Jewish Health).
SAE Reporting Plan: While we do not expect serious adverse events to occur, we will track and report any SAEs associated with study drug administration. The PIs will report any SAE and any decision to suspend or halt the protocol to the NJH IRB and to Gilead within 24-48 hours.
Protocol Stopping Criteria: Patients may be discontinued or withdrawn from the study for any of the following reasons:

- At the patient's request
- At the discretion of the Investigator, if deemed appropriate, for any reason
- At the discretion of the Sponsor, if deemed appropriate, for any reason
- IV antibiotic therapy is initiated during study.

If for any reason a patient does not complete the study, the reason will be entered on the CRF. All patients are free to withdraw from participation at any time, for any reason, specified or unspecified, and without prejudice. The PI must record the reason for the early termination.

3.4. Benefits:

Study subjects whose FEV1% predicted is between 25-75% will receive one month of inhaled Cayston® during enrollment in this study with the Altera® nebulizer for administration. The study has the potential to ultimately benefit patients with CF, as the lack of sensitive biomarkers is a major limitation in drug development for CF. The identification of more sensitive biomarkers could result in more therapies being available to individuals with CF and in broadening indications for currently available therapies which have not been able to show significant benefit in certain CF patient populations. In addition, sensitive biomarkers could allow for a more "personalized" approach to CF therapies, as effects on particular individuals could be demonstrated rapidly within the individual. In addition, subjects will not have the co-pays or deductibles normally associated with a one-month prescription of this medication, and the cost for a one-month prescription will not be billed to subjects, their insurance, or other third parties.

4. MATERIALS AND METHODS

4.1 Subjects

Subject Recruitment and Selection

Subjects enrolled in this proposal will be recruited from patients followed by the Adult CF Program at National Jewish Health. We project enrolling 35 subjects, with CF ≥ 18 years of age at starting a month long course of Cayston®, following a one month time period without the use of anti-pseudomonal inhaled antibiotics. This will allow for a 15% dropout rate, leaving 30 subjects completing the study. Concomitant use of systemic steroids will not be allowed, and typical co-infections or co-morbidities will not result in exclusion.

Informed Consent Procedure

Informed consent will be obtained prior to any study related procedures taking place or PHI being collected. When patients are identified by clinic staff as potential subjects for this study, the Research Coordinator will meet with the patient, explain the study design and procedures, and review inclusion and exclusion criteria. If the patient meets criteria and is interested in participating, informed consent will be obtained by the P.I., the Co-P.I, or the research coordinator.

1. A signed and dated copy of the consent form will be provided to the patient.
2. The protected health information (PHI) collected for this study will only be the patient's name and telephone numbers. This will be used to contact patients for their end-of-treatment follow-up appointment (Day 28 +/- 3). This PHI will be kept in a study specific regulatory binder in a secure and locked office. All other information collected for this study will be de-identified by using a study number.

Inclusion Criteria:

1. Documented diagnosis of CF.
2. Age 18 years old or greater.
3. FEV_1 % predicted greater than 25%
4. Ability to perform reproducible Pulmonary Function Tests.
5. Willingness to comply with study procedure and willingness to provide written consent.
6. Clinically stable without evidence of acute upper or lower respiratory tract infection or current pulmonary exacerbation within the 14 days prior to the screening

7. Ability to produce at least 1mL of sputum spontaneously, or be willing to undergo sputum induction
8. Chronic bacterial colonization with Pseudomonas aeruginosa (2 documented positive cultures in the prior 2 years.)
9. Patient is starting on a cycle of Cayston as part of their clinical care.

Exclusion Criteria:

1. Presence of a condition or abnormality that, in the opinion of the Principal Investigator (PI), would compromise the safety of the patient or the quality of the data.
2. Aztreonam allergy, bronchospasm, or other contraindication to use of aztreonam.
3. Signs and symptoms of acute pulmonary exacerbation at the time of enrollment or during study, requiring oral or intravenous antibiotic therapy.
4. Active infection and treatment for non-tuberculous mycobacteria.
5. Concomitant use of systemic steroids.
6. Use of inhaled antimicrobial agents with activity against *Pseudomonas aeruginosa* within 28 days prior to Visit 1.

4.2 Enrollment and Study Initiation Time Window

All patients will be enrolled following a screening visit to determine eligibility. After obtaining a signed and dated informed consent, each subject will be assigned a study ID number. For the purpose of this study, the study ID number will be used to identify the sample and subsequent clinical data.

4.3 Treatment with Cayston

Following enrollment, all subjects will be treated for clinical purposes with one month of Cayston® as it is FDA approved, 75 mg nebulized three times daily, using an Altera® nebulizer. For subjects whose FEV1% predicted is between 25-75%, the medication and nebulizer will be disbursed by the study coordinator and patients will be counseled to keep used respule containers to return at the end of the study. This one-month of drug will be provided by the sponsor. For those patients with an FEV1>75% predicted (technically off-label use) who are prescribed Cayston® by their treating CF physician, they will be approached for enrollment but will not be disbursed Cayston or the Altera nebulizer by the study coordinator, but through the prescription written by their treating MD.

4.4 Informed Consent

Primacy of Subject: All subjects have the final authority in decision-making and they cannot be overridden.

Protecting Computer Data: The PHI will be kept in a secure and locked location. Any computer data will be kept in password locked computers. All other information collected on the case report form and shared with oversight agencies will be de-identified with a subject code used.

4.5 Minorities and Women

Patients that meet the study criteria will be recruited without regard to gender and without specific exclusion of any national or racial group. Thus the proportions of women and minorities in the study population will closely mirror the proportions of these groups among adult CF patients in the Denver metropolitan area. While CF is distributed evenly between males and females, the genetic mutation that causes CF is most common in individuals of Northern European decent, and it is likely our study population will reflect this pattern. The patient population will consist exclusively of adults (age > 18 years).

4.6 Study Assessments
Background Assessments

1. Demographic information (age, gender, ethnicity)
2. CF diagnostic history (sweat test results, CF genotype),
3. History of CF-related medical conditions (meconium ileus, pancreatic status, CF-related diabetes, ABPA, CF-related liver disease)
4. Number of pulmonary exacerbations over the past year requiring antibiotics
5. Hospitalizations in the preceding year
6. Medications being taken within the preceding 14 days, during the study, and use of inhaled antibiotic therapy in the year preceding the study.
7. Sputum microbiology results of the preceding year

Baseline Assessments (Day 0-1)

1. History for current clinical status
2. Physical examination
3. Oxygen saturation (room air)
4. Sputum for bacterial identification (collected by spontaneous expectoration)
5. Spirometry and CRP and CBC
6. Blood sample (10 cc) for RNA isolation from whole blood.
7. Sputum IL-8, neutrophil elastase and serum IL-8.
8. CFQ-R symptom scale.

Assessments at study end (Day 28+/-3)

1. History and physical examination
2. Medications prescribed for the duration of the study
3. Oxygen saturation (room air)
4. Sputum for bacterial identification (collected by spontaneous expectoration)
5. Spirometry and CRP and CBC
6. Blood sample (10 cc) for RNA isolation from whole blood.
7. Sputum IL-8, neutrophil elastase and serum IL-8.
8. CFQ-R symptom scale.

4.6 Laboratory Assay Methods

Blood obtained for gene expression analysis (5 cc) will be processed with centrifugation in 2 PAXgene™ Blood RNA Tubes, which contain an additive that reduces in vitro RNA degradation and minimizes gene induction. The blood will be drawn in replicates, for the direct isolation of mRNA from whole blood. Briefly blood will be incubated at room temperature for 4 h for RNA stabilization and then stored at − 80 °C. RNA will be extracted from whole blood using the PAXgene™ Blood RNA System Kit following the manufacturer's guidelines. Following a 2-hour incubation at room temperature for 2 hours to ensure complete lysis, the tubes will be centrifuged for 10 min at 5,000×g, supernatant discarded and 500 µL of RNase-free water added to the pellet. After resuspension, centrifugation for 10 min at 5000×g and removal of the supernatant, the pellet will be resuspended in 350 µL of buffer BR1 and further purification of RNA completed following the manufacturer's protocol with on-column DNase digestion. RNA concentrations are determined using a NanoDrop® ND-1000 spectrophotometer (NanoDrop Technologies, Wilmington, DE). First strand cDNA is then made from 1 µg total RNA using a QuantiTect® Reverse Transcription Kit (Qiagen, Valencia, CA). The 10 gene panel is then assayed by real-time polymerase chain reaction (RT-PCR) using Sybrgreen® indicator on a 7300 Real Time PCR system, as previously described[30]. The primary outcome measure will be change in the CF 10 gene panel with Cayston® therapy and in combination with FEV_1, CRP, CBC,

and sputum neutrophil products. Sputum IL-8 and neutrophil elastase will be assayed as described previously, following processing with sputolysin[31]. Serum IL-8 will be quantified by ELISA from an additional 5mls of whole blood.

Laboratory Methods: 5 mLs of whole blood will be collected at **Visits 1 and 2** directly into 2 PaxGene tubes. The PaxGene tubes may be frozen for up to several months without loss of RNA quality, allowing samples to be batched for PCR. RNA and cDNA purification from whole blood is an automated process using Qiagen® spin column kits on a QIAcube, following a centrifugation and wash step. An additional 5 mls of whole blood will be collected at Visits 1 and 2 for serum IL8 quantification.

4.7 Statistical Methods

Statistical methods and data analysis: All patients who successfully provide blood samples for both time points will be analyzed. Patients who do not complete collection points will not be included in the analysis, nor will patients who develop acute exacerbations during their Cayston® month. As a reference, the recent study dropout rate in CF biomarker study was 5%.

Primary endpoints: 1) Circulating leukocyte transcripts by quantitative RT-PCR before and after 4 weeks Cayston® from whole blood.

Secondary endpoints: 1) Change in pulmonary function (FEV_1) before and after Cayston®. 2) Change in bacterial density (*P. aeruginosa* strains) before and after Cayston®. 3) Change in CRP, CBC, serum IL-8, sputum IL-8 and neutrophil elastase, and CFQ-R respiratory domain score before and after Cayston®. 4) Time to next pulmonary exacerbation (compared to average time between exacerbations in the year prior to initiating Cayston®) 5) Correlation of genes with 1), 2), 3), 4).

Analysis plan:

Primary outcome: The outcome measures for the primary endpoint are changes in individual gene transcripts and various combinations of gene transcripts, with the goal of determining significant changes [illegible]fore and after Cayston® therapy. The gene transcripts will be evaluated as continuous variables. Paired t-[illegible]ts will be used to measure changes (exploratory) in the gene transcripts. Next, we will assess variability of gene transcripts and combinations using paired logistic regression in order to determine which combination is most sensitive to changes in clinical status and improvement with therapy. The primary objective is to determine whether changes in gene markers (either singly or in combination) predict reduction of infection and inflammation following 4 weeks of Cayston® therapy. Receiver Operating Characteristic (ROC) curves from the regression models will quantify the sensitivity and specificity of the individual markers and combinations. Differences between CRP will be evaluated for significant changes, while circulating leukocyte differentials will not, since to date, neutrophil, lymphocyte, and monocyte counts have not changed significantly between blood draws spaced 2-3 weeks apart at the time of exacerbations. FEV_1 will be modeled as a function of gene expression (continuous variable) and visit (1 and 2). The relationship between changes in genes and changes in FEV_1, CRP, CBC, CFQ-R scores, sputum IL-8, serum IL-8, neutrophil elastase and bacterial density between visits 1 and 2 will be estimated from this model, and covariates, such as gender and comorbidities will be tested for significance in the model. Any non-normal outcomes will be transformed before analysis. A logistic regression analysis will be performed to test if patients with no or limited previous Cayston® exposure had a greater response than patients with over 3 cycles of Cayston® in the past 12 months.

Secondary outcomes: All pre and post changes in secondary measures (FEV_1, CRP, CBC, CFQ-R score, serum IL-8, sputum IL-8 and neutrophil elastase, bacterial density in log_{10}CFU/ml sputum) will be summarized using descriptive statistics. We will correlate changes in individual genes and groups of genes with secondary measures, using Pearson's correlation coefficient or Spearman's rank if data nonlinear.

Power calculations: Based on preliminary data (n=60), changes in the 10-gene panel range from 10% to 1000% in response to treatment for a pulmonary exacerbation, with a median change of 100%. Intra-assay and intra-individual biological variation for a single draw is negligible. It is therefore reasonable and conservative to assume that we will find a 60% response level. Based on preliminary data, differences in mean expression pre- and post- antibiotics ranged between 0.2 to 2 standard deviations, with ¾ of subjects <1 SD. Thus, using conservative estimates, the number of patients per group necessary for a 0.05 significance level paired t test to have 80% power for detecting a difference is n=28. Assuming a 10% dropout rate, we intend to recruit at least 30 patients in order to have 80% power to detect clinically significant changes in gene expression.

References

1. Accurso FJ. Update in cystic fibrosis 2005. Am J Respir Crit Care Med 2006;173:944-7.

2. Khan TZ, Wegner JS, Bost T, Martinez J, Accurso FJ, Riches DWH. Early Pulmonary Inflammation in Infants with Cystic Fibrosis. Am J Respir Crit Care Med 1995;151:1075-82.

3. Noah TL, Black HR, Cheng PW, Wood RE, Leigh MW. Nasal and bronchoalveolar lavage fluid cytokines in early cystic fibrosis. J Infect Dis 1997;175:638-47.

4. Birrer P, McElvaney NG, Rudeberg A, et al. Protease-antiprotease imbalance in the lungs of children with cystic fibrosis. Am J Respir Crit Care Med 1994;150:207-13.

5. Hull J, Vervaart P, Grimwood K, Phelan P. Pulmonary oxidative stress response in young children with cystic fibrosis. Thorax 1997;52:557-60.

6. Walker TS, Worthen GS, Poch KR, et al. Enhanced Pseudomonas aeruginosa biofilm development mediated by human neutrophils. Infect Immun 2005;73:3693-701.

7. Konstan MW, Byard PJ, Hoppel CL. Effect of high-dose ibuprofen in patients with CF. N Engl J Med 1995;332:848-52.

8. Ordonez CL, Henig NR, Mayer-Hamblett N, et al. Inflammatory and Microbiologic Markers in Induced Sputum following IV Antibiotics in Cystic Fibrosis. Am J Respir Crit Care Med 2003.

9. Durieu I, Peyrol S, Gindre D, Bellon G, Durand DV, Pacheco Y. Subepithelial fibrosis and degradation of the bronchial extracellular matrix in cystic fibrosis. Am J Respir Crit Care Med 1998;158:580-8.

10. Hubeau C, Lorenzato M, Couetil JP, et al. Quantitative analysis of inflammatory cells infiltrating the cystic fibrosis airway mucosa. Clin Exp Immunol 2001;124:69-76.

11. Corey M, Edwards L, Levison H, Knowles M. Longitudinal analysis of pulmonary function decline in patients with cystic fibrosis. J Pediatr 1997;131:809-14.

12. Cystic Fibrosis Foundation Patient Registry

2006 Annual Data Report to the Center Directors. In. Bethesda, MD: © 2007 Cystic Fibrosis Foundation.

13. Ramsey BW. Outcome measures for development of new therapies in cystic fibrosis: are we making progress and what are the next steps? Proc Am Thorac Soc 2007;4:367-9.

14. Katz R. Food and Drug Administration regulation. CNS Spectr 2008;13:45-6.

15. Rutherford RM, Kehren J, Staedtler F, et al. Functional genomics in sarcoidosis--reduced or increased apoptosis? Swiss Med Wkly 2001;131:459-70.

16. Hakonarson H, Bjornsdottir US, Halapi E, et al. Profiling of genes expressed in peripheral blood mononuclear cells predicts glucocorticoid sensitivity in asthma patients. Proc Natl Acad Sci U S A 2005;102:14789-94.

17. Ning W, Li CJ, Kaminski N, et al. Comprehensive gene expression profiles reveal pathways related to the pathogenesis of chronic obstructive pulmonary disease. Proc Natl Acad Sci U S A 2004;101:14895-900.

18. Whitney AR, Diehn M, Popper SJ, et al. Individuality and variation in gene expression patterns in human blood. Proc Natl Acad Sci U S A 2003;100:1896-901.

19. Saavedra MT, Hughes GJ, Sanders LA, et al. Circulating RNA transcripts identify therapeutic response in cystic fibrosis lung disease. Am J Respir Crit Care Med 2008;178:929-38.

20. Moller HJ, Moestrup SK, Weis N, et al. Macrophage serum markers in pneumococcal bacteremia: Prediction of survival by soluble CD163. Crit Care Med 2006;34:2561-6.

21. Wu Q, Martin RJ, Lafasto S, et al. Toll-like receptor 2 down-regulation in established mouse allergic lungs contributes to decreased mycoplasma clearance. Am J Respir Crit Care Med 2008;177:720-9.

22. Kim SH, Han SY, Azam T, Yoon DY, Dinarello CA. Interleukin-32: a cytokine and inducer of TNFalpha. Immunity 2005;22:131-42.

23. Netea MG, Lewis EC, Azam T, et al. Interleukin-32 induces the differentiation of monocytes into macrophage-like cells. Proc Natl Acad Sci U S A 2008;105:3515-20.

24. Rodrigo WW, Jin X, Blackley SD, Rose RC, Schlesinger JJ. Differential enhancement of dengue virus immune complex infectivity mediated by signaling-competent and signaling-incompetent human Fcgamma RIA (CD64) or FcgammaRIIA (CD32). J Virol 2006;80:10128-38.

25. Doyen V, Rubio M, Braun D, et al. Thrombospondin 1 is an autocrine negative regulator of human dendritic cell activation. J Exp Med 2003;198:1277-83.

26. Ferreira V, van Dijk KW, Groen AK, et al. Macrophage-specific inhibition of NF-kappaB activation reduces foam-cell formation. Atherosclerosis 2007;192:283-90.

27. Kwok CF, Juan CC, Ho LT. Endothelin-1 decreases CD36 protein expression in vascular smooth muscle cells. Am J Physiol Endocrinol Metab 2007;292:E648-52.

28. Weiss M, Schneider EM. Soluble CD163: An age-dependent, anti-inflammatory biomarker predicting outcome in sepsis. Crit Care Med 2006;34:2682-3.

29. Vaday GG, Lider O. Extracellular matrix moieties, cytokines, and enzymes: dynamic effects on immune cell behavior and inflammation. J Leukoc Biol 2000;67:149-59.

30. Saavedra MT, Hughes GJ, Sanders LA, et al. Circulating RNA transcripts identify therapeutic response in cystic fibrosis lung disease. Am J Respir Crit Care Med 2008;178:929-38.

31. Laguna TA, Wagner BD, Luckey HK, et al. Sputum desmosine during hospital admission for pulmonary exacerbation in cystic fibrosis. Chest 2009;136:1561-8.
